# Atypical tool and object use in wild immature chimpanzees reveals developmental pathways to innovation

**DOI:** 10.1038/s41598-025-20487-8

**Published:** 2025-10-17

**Authors:** Iulia Bădescu, Luseadra J. McKerracher, Daniel W. Sellen, David P. Watts, Felix Riede

**Affiliations:** 1 Département d’Anthropologie, Université de Montréal, Montréal, QC Canada; 2 Department of Public Health, Aarhus University, Aarhus, Denmark; 3 Department of Anthropology, University of Toronto, Toronto, ON Canada; 4 Department of Anthropology, Yale University, New Haven, CT USA; 5 Department of Archaeology and Heritage Studies, Aarhus University Moesgård, Aarhus, Denmark

**Keywords:** Innovation, Chimpanzees, Object play, Tool use development, Exploration and atypical behavior, Cultural evolution, Animal behaviour, Biological anthropology, Anthropology, Cultural evolution, Behavioural ecology, Archaeology

## Abstract

Innovation drives cultural evolution, yet little is known about its developmental origins or the role of immature individuals in generating novel behaviors. We examined 67 object uses by 36 infant and juvenile chimpanzees (*Pan troglodytes schweinfurthii*), over 15 months at Ngogo, Uganda. Nearly half of object uses were atypical, deviating from adult norms. 94% of atypical bouts were novel additions or modifications of adult forms or uses in new contexts, including three efficacious innovations: doll play, moss-sponging, and leaf-clipping to request carrying. To assess individual differences, we developed an Exploration Index integrating frequency, diversity, and atypicality of object use. Nine individuals had notably higher scores. Females and offspring of multiparous mothers scored higher, indicating effects of sex and possible social scaffolding from experienced mothers and siblings on object use. These findings suggest that immatures generate novelty at the margins of species-typical behavior yet vary in their propensity to innovate. A permissive social environment for object play may be key to the developmental pathways of innovation, providing a generative context for behavioral variation on which social learning and selection can act. If retained and transmitted, even rare innovations by immatures could contribute to the accumulation of cultural complexity.

**Supplementary Information:**

The online version contains supplementary material available at 10.1038/s41598-025-20487-8.

## Introduction

Behavioral innovation and high-fidelity social transmission underpin human cultural and technological complexity^1^. The capacity for cumulative cultural evolution—the incremental refinement, transmission, and maintenance of technological and social innovations over generations—has been instrumental in achieving this complexity, and is often argued to be a defining feature of the genus *Homo*^2–4^. That said, the timing of the emergence of the capacity for cumulative cultural evolution within the Primate order remains debated^5,6^. Indeed, experimental and observational evidence from chimpanzees and other non-human primate taxa suggests that social learning of behaviors from conspecifics that then persist and accumulate within populations to form traditions or ‘cultures’ may be common^7–11^, with the main differences between humans and our relatives being ones of mechanism (i.e., high-fidelity imitation versus other forms of copying) and ones of scale (i.e., frequency and rapidity of behavioral experimentation, invention and diffusion)^12^.

Understanding the ultimate pathways enabling cumulative cultural evolution requires investigating how flexible, exploratory behaviors that lead to innovations emerge in other primate taxa and especially in our closest living relatives^2,12,13^. Chimpanzees (*Pan troglodytes*) offer valuable insights, as they exhibit the most complex and diverse repertoire of cultural traditions documented in the wild of any non-*Homo* species^8,9,11,14^. Key examples of well-documented chimpanzee traditions include community-specific tool use like leaf-sponging, termite fishing and nut-cracking, and social conventions such as grooming hand-clasps, leaf-grooming and leaf-clipping^8–11,14–17^.

While most relevant research on the generation of novel behaviors in wild chimpanzees has focused on adults, immature individuals may play key roles in behavioral innovation^1,18–22^. Innovations comprise behaviors that are novel in form and/or context (i.e., not strictly copied from others) and are functionally appropriate (following definitions by^1,21,23^). Infants and juveniles engage in high rates of play, exploration, and object manipulation, which build cognitive and motor skills and may lead to the emergence of novel behaviors that can include innovations^18,20,24–32^.

The mechanisms through which novel behaviors emerge and spread within a community or population are debated^2,12,30,33,34^. The Zone of Latent Solutions hypothesis (ZLS)^2,31^ posits that nonhuman animals, including chimpanzees, socially acquire behaviors that lie within their species-specific repertoire of individually (re)inventable actions, without high-fidelity imitation. In this context, behavioral variants arise from repeated independent learning, whereas cumulative cultural evolution requires a uniquely human capacity to copy form or “know-how.” Empirical and conceptual critiques have challenged the logic, testability, and ecological realism of the ZLS^5,12,34,35^. In particular, a growing body of work shows that chimpanzees can and do acquire multi-step behaviors through social learning^35–37^. This has led to the proposal of a Zone of Proximal Acquisition (ZPA), which conceptualizes the set of behaviors an individual is developmentally and cognitively prepared to acquire at a given time, through trial-and-error or social learning, depending on prior experience, social context, and environmental opportunity^12,34^. The central differences between these two frameworks concern the spread and maintenance of behaviors within a community or population, but they appear to share common threads regarding sources of behavioral novelty and variation. That is, while neither the ZLS nor the ZPA frameworks of cultural evolution explicitly specify which members of a population are most likely to innovate, at what ages or developmental stages innovation is most likely, or which socio-ecological conditions promote or suppress innovation, both frameworks emphasize the cognitive flexibility and developmental plasticity underlying innovation^2,12,34,38,39^—features characteristic of infancy and juvenility^40^.

Immature chimpanzees and other primates often manipulate leaves, sticks, stones, and other natural materials during play and while they are engaged in seemingly non-functional environmental exploration that they may refine into goal-directed actions^22,32,41–45^. We refer to these behaviors as *object use*. Like human children^46^, infant and juvenile chimpanzees experience a highly socially permissive developmental window, marked by high tolerance from other group members. Here, we propose a developmentalist perspective on the ZLS and ZPA frameworks in which this socially permissive window enables chimpanzee infants and juveniles to engage in atypical, unusual or exploratory object use, which can introduce guided variation—non-random behavioral modifications shaped by developmental, social, and ecological influences—into the pool of material culture use and may occasionally lead to novel, functional behaviors.

We examined tool use, object play and exploration among immature wild chimpanzees in the Ngogo community of Kibale National Park, Uganda. With a focus on investigating assumptions shared between the ZLS and the ZPA pertaining to sources of behavioral variation, we asked whether infant and juvenile chimpanzees from this community use objects in ways that diverge from typical adult use, and whether some individuals exhibit consistently high object use and exploration—potential precursors to innovation. We highlight instances of novel or functionally meaningful object use that may reflect innovations. We also investigated whether object-directed behavior varies as a function of infant and juvenile sex or maternal parity, although neither the ZLS or the ZPA specifically predicts such variation, because prior empirical evidence points towards sex and parity biases in object use and in social permissiveness. That is, adult female chimpanzees rely more on tool-based foraging than males do^7,47–49^; older, more experienced mothers may provide greater opportunities for object interaction through frequent modeling and higher permissiveness; and, the presence of older siblings may foster opportunities for object play^46,50–54^.

## Results

Over 15 months in 2013–2014 and 2018, the lead author observed 67 object use bouts by 36 immature chimpanzees during focal follows and *ad libitum* sampling (Table 1). Object use occurred across a broad age range (0.5 to 13 years), although it was most common among infants aged 1–3 years old (47/67 cases; 70.2%). The number of object use bouts per individual ranged from 1 to 5 (Mean = 1.86, SD = 1.13), with 16 individuals (44%) contributing more than one bout. Most individuals were observed to use objects at a single age (in years), while four individuals (11%) used objects across more than one age.

**Table 1:** Observed object use by immature chimpanzees at Ngogo, Kibale.

Object use category (no. of bouts)	Ages (years)	Frequency	Proportion of total (%)
Tool	0.5 to 13	28	41.8
* Leaf-clipping (1)*	*0.5*		
* Leaf-grooming (8)*	*0.5 to 7*		
* Leaf napkin (1)*	*3*		
* Sponge (12)*	*2 to 9*		
* Stick tool*,* honey dipping (2)*	*2 and 13*		
* Stick tool*,* insect fishing (2)*	*1*		
* Stick tool*,* drink water attempt (2)*	*2 and 3*		
Doll	1	2	3.0
Aggression	2 to 4	7	10.4
* Branch shake (4)*	*2 to 4*		
* Stick throw (3)*	*2*		
Game	1 to 9	11	16.4
Locomotion aid	4 to 8	2	3.0
Physical stimulation	1 to 6	8	11.9
* Scratch body (5)*	*1 to 6*		
* Masturbate (3)*	*1 to 6*		
Play nest	2 to 3	9	13.4
**TOTAL**	**0.5 to 13**	**67**	**100**

Object use spanned seven categories. Tool use was most frequent (41.8%) and included tools used in social contexts (i.e., leaf-clipping, leaf-grooming), hygiene (i.e., leaf napkin), and extractive foraging (i.e., stick tools for honey dipping and insect fishing; leaf sponge to drink water). Games with objects accounted for 16.4% of interactions, and included, for example, ‘keep-away’ games where one individual withheld a stick from another individual who repeatedly tried to get it. Other object interactions included making and using a nest during social or solitary play (13.4%), physical stimulation to scratch skin after rains, rubbing sticks on genitalia (‘masturbation’; 11.9%), and aggression (branch shaking by one infant toward another; stick throwing during bipedal displays; 10.4%). Rare object uses included use of vegetation to make ‘bridges’ between trees (locomotion aid, 3.0%; for example, juvenile OF connected the branches of two trees to make it possible for his infant sister, NA, to cross from one tree to the other) and two ‘doll play’ bouts, each lasting a few hours, in which 1-year-old female CF carried a tree stump on her ventrum and back, mimicking adult infant-carrying.

### Typical, atypical, and innovative object uses

About half (50.8%) of the object and tool uses were done in typical adult ways, while 49.3% were atypical—that is, either different from adult uses or not seen among adults at Ngogo. Of the 33 atypical object interactions, only 2 (6.1%) were *omission uses*, where infants attempted typical adult behavior but missed key steps or components. For example, after intense peering at her mother and siblings ‘leaf-grooming’ during social grooming, 1-year-old CH held a leaf very close to her eyes and stared at it for a prolonged period. She did not manipulate the leaf with her fingertips or lips—key components leaf-grooming^14^. This was interpreted as an attempt at leaf-grooming by CH, but lacking the defining motor elements, it was categorized as an *omission use*.

The other 31 (93.9%) atypical object interactions were *commission uses*, where infants engaged with objects in unconventional or unexpected ways, modifying or adding actions, or using an established tool in a new context, that differed from the typical, adult use (e.g., dipping stick into water to attempt drinking). Younger immature chimpanzees (0.5–4 years) used objects atypically more often (53.6% of bouts) than older immature individuals (> 4 years; 27.3% of bouts), but the difference was not significant by conventional standards (Logistic regression: β = − 0.20, *p* = 0.08), especially after adjustment for sex and maternal parity (Huber’s M-estimator, β = − 0.28, *p* = 0.11; Table 2).

**Table 2:** Robust regression (Huber’s M-estimator) results for factors influencing individual exploration Indexes.

Predictor	Coefficient	Std. error	z-value	*p*-value	95% CI (Lower, Upper)
Intercept	–0.46	2.31	–0.20	0.841	–4.99, 4.07
Sex (male)	–2.23	1.09	–2.05	**0.040**	–4.36, -0.10
Parity (multiparous)	2.66	1.25	2.12	**0.034**	0.20, 5.12
Central age	–0.28	0.18	–1.58	0.114	–0.62, 0.07

Most of the *commission* object uses were likely ineffective or impractical. For example, infants HD and TO modified sticks and attempted to use them as tools for drinking by dipping them into water, but this failed to transfer liquid. Some *commission* uses proved both innovative and efficacious, however. Notable examples included: (1) Two cases of ‘doll play’ described above. (2) One case of using a clump of moss as a sponge to soak up and drink water by six-year-old female NA, who thereby bypassed the need to process leaves into a leaf-sponge. As moss is naturally absorbent and is readily available at Ngogo, this method was likely more efficient (holding a greater volume of water) and effective (taking less time to manufacture and use) than the typical leaf-sponge method, as shown experimentally in another chimpanzee community^55^. (3) One case of using the leaf-clipping gesture in a new context by one-year-old male KE to request to be carried by his mother—a signal that worked, as she returned, presented her rump, and allowed KE to climb onto her back. Approximately 90% of leaf-clipping at Ngogo is done by adult males to solicit copulations from estrus females, with rarer cases of leaf-clipping by estrus females before copulation, by adult males before dominance displays and before hunting of red colobus, and by a juvenile male during social play^16^. Thus, KE repurposed the leaf-clipping gesture for a novel, infant-specific use.

### Individual differences and predictors of object use

**Fig. 1:**
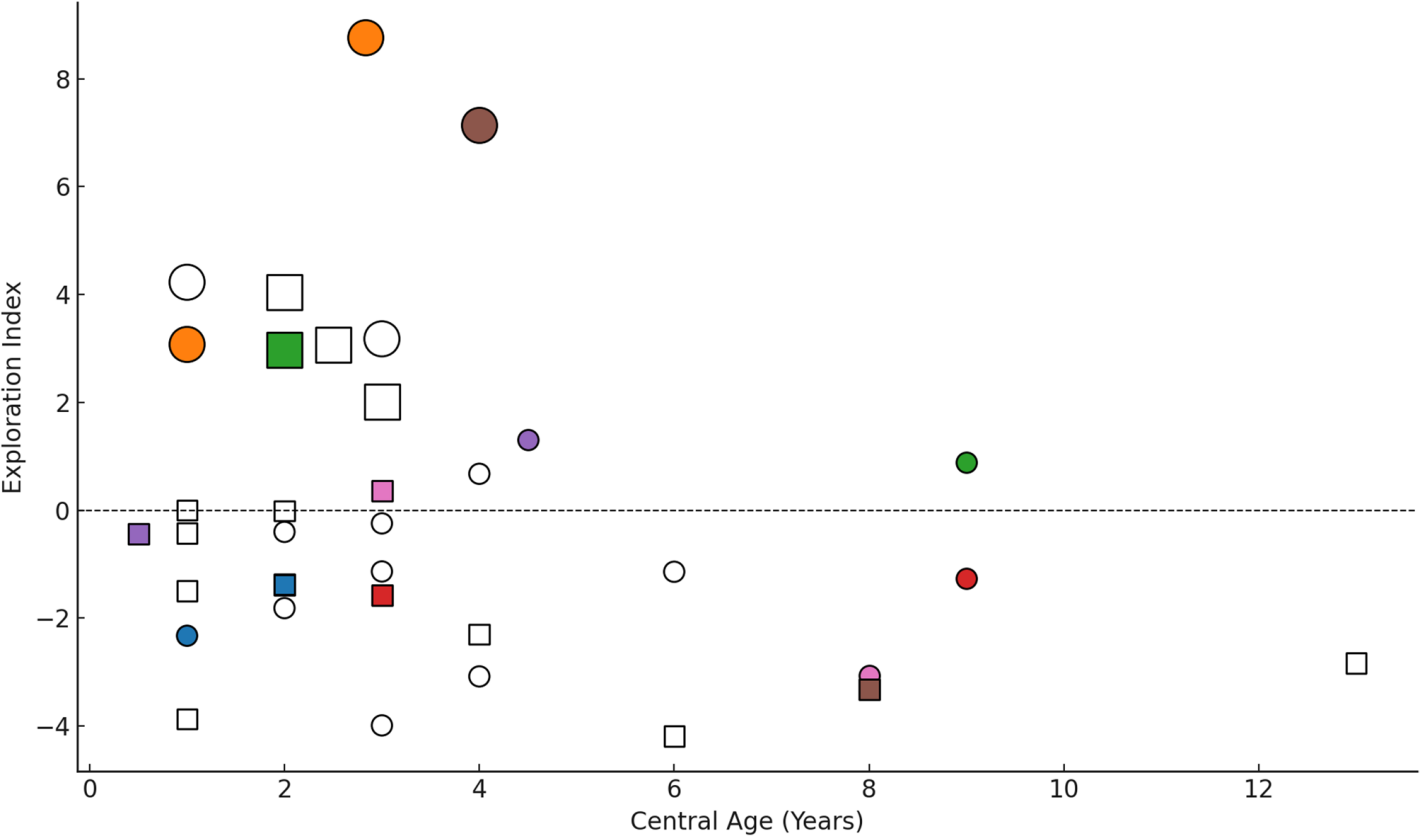
Individual Exploration Index versus Central Age differentiated by K-means cluster type (large symbols = high explorers, small symbol = low explorers), Sex (circles = females, squares = males) and Sibling Group (filled markers by color = individuals belonging to one of seven sibling groups; no fill markers = individuals without siblings in the dataset).

We used (1) the total number of object interactions, (2) object diversity (number of unique types used), (3) proportion of atypical/typical object use, (4) individual observation hours, and (5) age diversity (number of age categories—0–1, 1–2, 2–3 years, etc.—during which object use was observed) to calculate an Exploration Index for each infant and juvenile. A k-means clustering analysis of the Exploration Indexes revealed two distinct groups among the chimpanzee infants and juveniles (Fig. 1). High explorers (5 females: CF, CH, JO, KO, NA; 4 males: BB, CL, TO, ZW) had higher index values (mean = 4.28, SD = 2.22, range = 2.02–8.77) than low explorers (13 females, 14 males; mean=-1.43, SD = 1.54, Range=-4.19-1.30; Mann-Whitney U: U = 243.0, *p* < 0.001). They demonstrated greater object use frequency and diversity and higher rates of atypical use, which reflects more interest in objects and greater tendencies for exploration and potential innovation. They also used objects over a greater age range, which indicates persistent exploration. Object use by low explorers resembled the normative behavior of adults, in that adults rarely manipulate objects in non-functional ways.

**Fig. 2:**
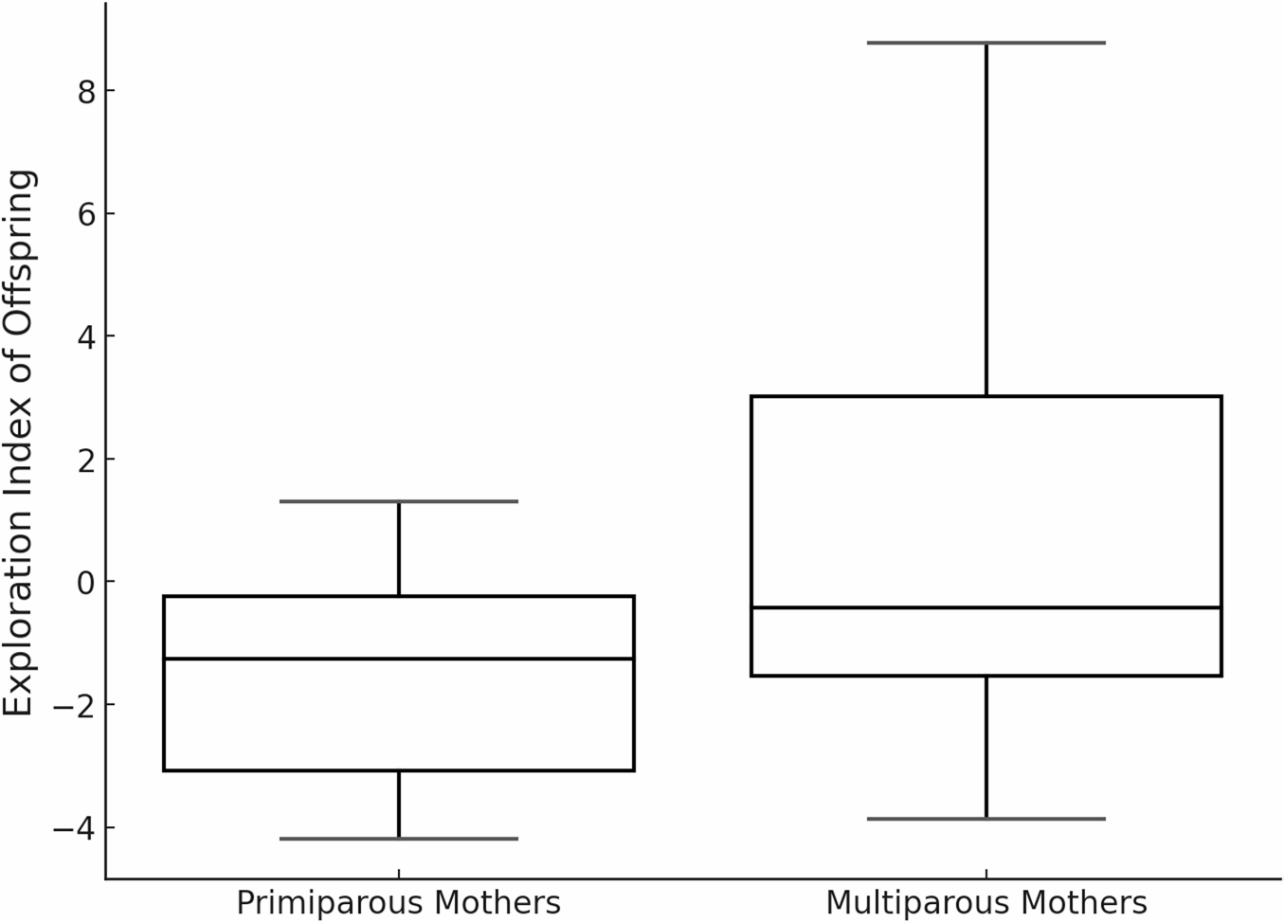
Box and whisker plot showing the distribution in immature chimpanzee exploration indexes by maternal parity. The box represents the interquartile range (IQR), with the median shown as a line inside the box. Whiskers extend to values within 1.5 × IQR.

Exploration Index scores varied by sex and maternal parity. Females had higher scores than males (Robust regression, Huber’s M-estimator: β = − 2.23, *p* = 0.040; Table 2; Fig. 1), and offspring of multiparous mothers had higher scores than those of primiparous mothers (β = 2.66, *p* = 0.034; Fig. 2). Exploration indexes were not significantly associated with the central ages of individuals (β = − 0.28, *p* = 0.114). The robust regression results showed the same direction and significance of effects as the initial ordinary least squares (OLS) model.

To assess whether maternal siblings (*N* = 7 pairs, Fig. 1) were more similar in their exploration behavior than non-siblings, we calculated the intraclass correlation coefficient (ICC) from a linear mixed-effects model with maternal ID as a random effect and no fixed predictors. The ICC was 0.10, indicating that ~ 10% of the variance in exploration scores was attributable to maternal identity. This suggests that maternal siblings were not more similar in their object use and exploration tendencies than expected by chance.

## Discussion

Infant and juvenile chimpanzees at Ngogo engaged relatively often with tools and other objects in ways that diverged from the community’s typical, adult forms. Moreover, some immatures were particularly likely to engage in exploratory object use and to do so in ways that could lead to innovation. Atypical *commission uses* that were novel, unconventional, or idiosyncratic manipulations accounted for nearly half of all observed object use. As expected, these behaviors were most common in immature females born to a multiparous mother and were concentrated among relatively motivated or explorative individuals who used objects more frequently, in more diverse ways and with greater atypicality. Most atypical object uses by immature chimpanzees were playful and seemingly ineffective, but three may meet criteria for innovations because they were individually generated, spontaneous, novel forms of behavior: doll play, moss-sponging to absorb and drink water, and the re-purposing of leaf-clipping as a request to be carried. The absence of these object uses in these contexts by other individuals of this community, and their rarity in our dataset suggest that they were spontaneous, individually-generated behavioral novelties that can be classed as innovations^1,21^. Our findings point towards a potentially important role for some immature chimpanzees as sources of behavioral novelty, in keeping with arguments that the exploratory behavior of immatures in many species that depend crucially on learning can lead to behavioral innovations^25,56^.

Identifying sources of behavioral variation and innovation in non-human primates is central to two main frameworks for understanding the form of, and capacity for, cumulative cultural evolution: the Zone of Latent Solutions (ZLS)^2,31^ and the Zone of Proximal Acquisition (ZPA)^12,34^. These frameworks are often seen as in conflict, especially in terms of the mechanisms responsible for diffusion and maintenance of cultural behaviors. However, both acknowledge that behavioral variation and innovation originate in individual learning, shaped by cognitive flexibility, access to raw materials and to social and ecological opportunities (including those related to sex, sibship order, maternal parity, and individual characteristics). Neither framework requires that variants must diffuse to validly constitute innovations. The behavioral innovation space in which immatures play with and explore objects in non-normative ways, falls within this shared theoretical territory. Our findings reveal the generative potential of this space. Immature primates are developmentally prone to explore and to invent novel behaviors in general^29,30,34,57,58^. We suggest that low social constraints and high intrinsic motivation during early development, combined with the high social permissiveness and developmental affordances of early life, offer a unique window enabling immature individuals to generate behavioral variation, even in the absence of explicit models or functional goals^25,29,30,34,46,56,59^.

Our findings may illustrate two pathways–each compatible with both the ZLS and the ZPA–through which behavioral innovations can arise in immature chimpanzees: through individual (re)invention of novel behaviors not requiring high-fidelity imitation, and through the modification or recontextualization of existing traditions^2,8,9,11,31,34,38,60^. In the first category, doll play that simulates infant-carrying by mothers had not previously been reported from Ngogo. It has been reported at the nearby Kanyawara site^61^, but the two cases we recorded at Ngogo, both performed by the same 1-year-old female, likely represented independent innovation. Doll play could serve a socio-cognitive developmental function, akin to play-based practice for future caregiving^61,62^.

In the second category, we observed innovations that built on or reconfigured behaviors in the community’s existing repertoire. For example, moss-sponging by a 6-year-old female involved using a novel substrate for an already-established form of tool-use at Ngogo (leaf-sponging). Moss-sponging could be a latent solution expressed through individual trial-and-error (as per ZLS)^31^, but was likely facilitated by prior exposure to leaf sponging by adults (as per ZPA)^34^. Similarly, leaf-clipping by a 1-year-old male repurposed a social tool primarily used in mating contexts to request maternal carrying. These cases presumably are evidence of cognitive flexibility and the ability to reconfigure existing behavioral elements in novel ways^12,34^.

Some novel behaviors can emerge and persist within peer groups of immatures, forming developmentally bounded ‘childhood cultures’^30,34,62^. These behaviors – in our case, possibly doll play or the leaf-clipping gesture to request carrying – may not be functionally relevant to extend into adulthood to become community-wide traditions but could reflect exploratory innovation with potential to become traditions in infant and juvenile social networks^62,63^. None of the innovations we observed appear to have spread to other individuals however, perhaps reflecting a persistent gap between the emergence of behavioral novelty, on the one hand, and the diffusion of novel behaviors, on the other, due to constraints on social transmission in nonhuman primates^20^, even when the novel behavior is potentially fitness-enhancing. The moss-sponging case illustrates this disconnect. Moss is more absorbent than leaves and has been shown experimentally to be more effective for obtaining water than leaf sponges^55^. It may also offer health benefits due to its antibacterial and antifungal compounds^64^. Despite these advantages, moss-sponging is not widespread at Ngogo. This contrasts with the moss-sponging innovation and subsequent uptake at the Sonso chimpanzee community in Budongo. Here, moss-sponging was innovated by the group’s alpha male and subsequently spread via social transmission^65^. The differences in the outcomes between the two sites illustrates how functionally-effective innovation alone is insufficient for cultural transmission^20^; rather, conformity pressure, conservatism in chimpanzee learning^66,67^, technological lock-in (i.e., preference for familiar tools and substrates^22,68,69^, and social factors such as kinship, rank and individual positions in social networks can shape whether new behaviors are noticed and copied^37,70,71^. Innovations by low-status, immature individuals in dyadic contexts (e.g. infant leaf-clipping request to be carried by mother) may go unnoticed or lack the social salience needed to spread^72–74^ (see^15,18^ for adult chimpanzee examples of innovations that also failed to diffuse).

Young females and offspring of multiparous mothers had higher Exploration Index scores than young males and offspring of primiparous mothers. These findings align with the novel behavioral variant components of both the ZLS and the ZPA and with previous empirical work on chimpanzee object use in that female chimpanzees acquire tool-use competencies earlier and use tools more frequently in adulthood^47–49,75^. Also, immature females showed more doll play at Kanywara^61^ and built day-nests at younger ages and more often at Ngogo^76^, suggesting that they are more motivated than young males to manipulate and use objects. This may reflect a broader developmental pattern in which chimpanzee females achieve independence from their mothers at earlier ages than males^77–80^. Also, multiparous mothers may allow offspring greater autonomy and offer more exploration opportunities than primipares^50,52,54^. Older siblings may be social models and catalysts for object use and exploration through object play, co-use of tools, or reduction of social inhibition^53,81–83^.

Our findings are subject to several limitations. Firstly, we did not compare immature and adult exploratory object use. Doing so would strengthen our claim that infants and juveniles are more prone than adults to varied and flexible object manipulations and explore a wider behavioral space in which they can innovate^29,30^. Secondly, our dataset included multiple observations of some individuals, but we could not track the extended developmental trajectories of most infants and juveniles. Consequently, we could not assess how individual object use and exploratory behavior changed across development, nor how early object play relates to later tool-use proficiency or innovation. Such a long-term perspective could clarify whether early *commission* object use or high exploration scores predict later competence, creativity, or tool use innovation. Future work should examine whether mothers’ own tendencies toward object use, as well as offspring preferences for specific materials or techniques, influence individual exploratory patterns. Lastly, some of the innovations we observed might have spread or have been first performed by adults and later repeated by immatures but went undetected due to inevitable limits on observation. Newly immigrant females could have introduced the innovations we observed, but this seems unlikely, because the innovators’ mothers were multiparous, long‑term members of the Ngogo community, and empirical evidence from Taï shows that immigrant chimpanzee females typically adopted local traditions rather than spreading those from their natal communities^67^.

Our findings support the view that exploratory object use in early life may serve as a generative context for behavioral flexibility and innovation^25,30,34,56,59^. Immature individuals may generate novel forms of behavior on which social learning, transmission mechanisms, and selection can subsequently act^1,15,43^. Immatures may be particularly important in otherwise conservative cultural systems such as those found in chimpanzees, where outliers can drive potential change^84,85^. The transient, individually-generated innovations that we observed in exploratory object play in low-risk, permissive settings in early life could provide a developmental substrate for cultural evolution^2,15,22,25,30,34,59^.

Beyond extant chimpanzees, this study has potential relevance for understanding the origins of tool use and cultural evolution in hominins. Prior to the emergence of cumulative cultural evolution featuring high-fidelity copying of specific manufacturing processes and forms^3^, early hominin tool use likely relied on repeated, sometimes socially-facilitated individual (re)invention and/or primed emulation of end products under variable ecological conditions^31,60,86^. Exploration in infancy and juvenility is supported by developmental features common across primates, including high neural plasticity, reduced social constraints, and strong intrinsic motivation to manipulate and interact with objects^22,24,26,46,59^. These traits may create a permissive window for discovering functional behaviors, especially when social learning cues are present but not prescriptive^2,12,31,34^. Conversely, the slow pace of cultural change across the first million or so years of hominin cultural evolution may attest to the relative absence of social contexts in which such permissive windows were open.

The non-transmitted innovations and other atypical, *commission* object uses observed in our study illustrate this developmental dynamic: individually generated, variably effective behaviors that potentially represent raw material for cultural traditions^8,14,87^. This may offer a model for how early hominin innovations emerged—through flexible and varied exploratory interactions with the environment, shaped by both individual experience (as per the ZLS hypothesis) and social-developmental scaffolding (as per the ZPA hypothesis). Over evolutionary timescales, even infrequent infant and juvenile-led innovations could accumulate culturally if retained and socially transmitted^86,88^.

Understanding these early-life pathways expands our perspective on the cognitive plasticity and behavioral scaffolding that underpins material culture in both extant apes and in the hominin lineage. Increased comparative and longitudinal attention to object manipulation by adults and immatures, and to the specific properties of the objects they manipulate, may allow future research to pinpoint instances of playful yet salient innovation. Comparative work on extant primates could provide insights into how playful innovation might have upscaled to the formation of lasting cultural traditions in hominin tool use, and how to recognize the archaeological signatures of such upscaling.

## Methods

### Study site and subjects

Ngogo lies in the center of Kibale National Park (KNP), western Uganda. KNP spans 795 km² (0°13′–0°41′ N, 30°19′–30°32′ E) and comprises a mix of moist evergreen and semideciduous forests, situated between lowland and montane forest zones^89^. The Ngogo study site consists of old-growth dry forest, with adjacent regenerating forest, swamp patches, and former anthropogenic grasslands^90^. Elevation ranges from 1400 to 1600 m, with average annual rainfall around 1479 mm, concentrated in March–June and September–November^89,91^. Chimpanzees at Ngogo have been studied since 1995 and are well habituated^92–95^. The community numbered 199–207 individuals during the years of this study, and their home range spanned approximately 35 km²^79,80,96^. Between the two study periods, the Ngogo chimpanzee community underwent a permanent fission, and two distinct communities now exist^97^. Although the chimpanzee toolkit at Ngogo is smaller than at other well-studied sites, tool use—primarily by adults—occurs in multiple contexts^16^. Tool-assisted extractive foraging is relatively uncommon and mostly involves leaf-sponging for water and use of sticks for honey-dipping. Tool-assisted foraging for insects is rare, probably in part due to the absence of termite mounds^16,98^. In contrast, hygiene-related tool use is common at Ngogo, with leaf napkins employed for cleaning wounds and body surfaces, such as genitalia after copulations^16^. Object use during social contexts, including leaf grooming and leaf clipping, are relatively common^16^. Tool use by infant and juvenile chimpanzees at Ngogo has been described as infrequent, but previous investigations did not focus on observing immatures; studies of adult tool use have centered on males, with comparatively less attention to females^16,98^.

### Data collection

Observations were conducted January–March 2013, September 2013–June 2014 and January–April 2018. We included 31 infants—defined as individuals who engaged in nipple and ventro-ventral contact with their mothers and lacked a younger sibling^78–80,99^—because they were observed using objects or tools. They ranged in age from 6 months to 6 years, although others have defined the infancy period as extending only to age 5^100^. We also included 5 juveniles (aged 4 to 13 years) who were observed to use objects; these individuals no longer made nipple or ventro-ventral contact, had at least one younger maternal sibling, and still traveled daily with their mothers^78–80,99^. One-hour focal animal sampling with continuous recording was conducted on infants, reflecting the original focus of the data collection on infant feeding and care during the lead author’s doctoral research. Object and tool use by juveniles was recorded opportunistically during *ad libitum* sampling^101^. We defined object use as any physical interaction with a natural item (e.g., leaf, stick, branch, moss) fitting into one of seven categories: tool use, ‘doll play’, aggression, game (i.e., object-centered play involving reciprocal or solo actions), locomotion aid, physical stimulation, or play nest (Table 1). We did not include the manipulation of food items while eating (e.g., investigating fruits or leaves while foraging). We identified distinct bouts of object use when individuals interacted with a new object. We obtained 569.58 h of contact time (mean: 14.99; range: 2.72–37.24) across the 36 infants and juveniles observed using objects.

### Analyses

Each object interaction was categorized as either typical, when behaviors followed established adult patterns (described by^16^) or atypical. Atypical object interactions were further divided into *omission uses*, which occurred when behaviors resembled typical use but lacked key steps or elements (terms and concept from a network cognition framework:^102^), and *commission uses*, when individuals used objects in new, unconventional ways, such as using a new technique, a new type of tool, or an established tool in a novel social or physical context. *Commission* object uses were evidence of exploratory or experimental behaviors that can lead to innovation^15,31^.

To quantify individual variation, we calculated an Exploration Index for each chimpanzee using a composite of five normalized metrics: (1) Total number of object use bouts; (2) Object use diversity (number of categories used); (3) Proportion of atypical uses (*commission* and *omission* *uses* relative to total); (4) Object use rate (bouts per observation hour); and (5) Age diversity (number of age points–0, 1, 2, 3, etc. yrs old–at which object use occurred). Each metric was z-transformed across individuals to standardize scale and ensure equal weighting. The overall Exploration Index was calculated as the sum of the z-scores. This composite measure captured broad exploratory tendencies rather than fine grained technical performance, allowing identification of relatively higher- and lower-exploring individuals even when some contributed only one bout to the dataset. We applied k-means clustering to the resulting Exploration Indexes to identify patterns of individual variation. The optimal number of clusters (k = 2) was determined using the Elbow Method^103^. A Mann–Whitney U test confirmed a significant difference between the two clusters (*U* = 0.0, *p* < 0.001).

To evaluate predictors of object exploration, we modeled the Exploration Index as a function of sex (female, male), maternal parity (primiparous, multiparous), and central age (individual’s mean age of object use). Sex and parity were treated as categorical predictors, and age as continuous. Although we initially fit an ordinary least squares (OLS) model, residual diagnostics indicated potential violations of normality and homoscedasticity. Therefore, we conducted a robust regression using Huber’s M-estimator, which is less sensitive to such violations, to assess the stability and significance of predictor effects. To assess whether maternal siblings had more similar exploration scores than non-siblings, we fit a linear mixed-effects model (LMM) with maternal ID as a random effect (no fixed predictors) and calculated the intraclass correlation coefficient (ICC) to estimate the variance in Exploration Index scores attributable to maternal identity. Statistical analyses were conducted with the assistance of ChatGPT (OpenAI), an AI language model. The tool was used to help design statistical approaches, interpret results, and generate code in Python using the *statsmodels* package. All analyses were verified and validated by the authors. Data analyzed are available in the Supplementary Information (Supplementary Data 1 and Supplementary Data 2).

## Supplementary Information

Below is the link to the electronic supplementary material.

Supplementary Material 1

Supplementary Material 2

## Data Availability

The datasets generated and analyzed in this study are included as Supplementary Data files.
